# A Case-Control Study Evaluating the Relationship Between Thimerosal-Containing *Haemophilus influenzae* Type b Vaccine Administration and the Risk for a Pervasive Developmental Disorder Diagnosis in the United States

**DOI:** 10.1007/s12011-014-0169-3

**Published:** 2014-11-11

**Authors:** David A. Geier, Janet K. Kern, Paul G. King, Lisa K. Sykes, Mark R. Geier

**Affiliations:** 1 The Institute of Chronic Illnesses, Inc, 14 Redgate Ct, Silver Spring, MD 20905 USA; 2 Department of Psychiatry, University of Texas Southwestern Medical Center at Dallas, Dallas, TX USA; 3 CoMeD, Inc, Silver Spring, MD USA

**Keywords:** Autism, Ethylmercury, Merthiolate, Thimerosal, Thiomersal, Vaccine

## Abstract

Thimerosal is an organic mercury (Hg)-containing compound (49.55 % Hg by weight) historically added to many multi-dose vials of vaccine as a preservative. A hypothesis testing case-control study evaluated automated medical records in the Vaccine Safety Datalink (VSD) for organic Hg exposure from Thimerosal in *Haemophilus influenzae* type b (Hib)-containing vaccines administered at specific times within the first 15 months of life among subjects diagnosed with pervasive developmental disorder (PDD) (*n* = 534) in comparison to controls. The generally accepted biologically non-plausible linkage between Thimerosal exposure and subsequent diagnosis of febrile seizure (*n* = 5886) was examined as a control outcome. Cases diagnosed with PDD received significantly more organic Hg within the first 6 months of life (odds ratio (OR) = 1.97, *p* < 0.001) and first 15 months of life (OR = 3.94, *p* < 0.0001) than controls, whereas cases diagnosed with febrile seizure were no more likely than controls to have received increased organic Hg. On a per microgram of organic Hg basis, cases diagnosed with a PDD in comparison to controls were at significantly greater odds (OR = 1.0197, *p* < 0.0001) of receiving increasing organic Hg exposure within the first 15 months of life, whereas cases diagnosed febrile seizure were no more likely than controls (OR = 0.999, *p* > 0.20) to have received increasing organic Hg exposure within the first 15 months of life. Routine childhood vaccination is an important public health tool to reduce the morbidity and mortality associated with infectious diseases, but the present study provides new epidemiological evidence of a significant relationship between increasing organic Hg exposure from Thimerosal-containing vaccines and the subsequent risk of PDD diagnosis in males and females.

## Introduction

Thimerosal is an organic mercury (Hg)-containing compound (49.55 % Hg by weight) historically added to many multi-dose vials of vaccine as a preservative since the 1930s [1]. Thimerosal is initially metabolized into ethyl-Hg compounds and thiosalicylate and rapidly binds onto thiol groups found on many proteins in human blood [2]. It is then actively transported across the blood brain barrier, including by the l-type neutral amino acid carrier transport (LAT) system, into human neuronal cells [3, 4], where it significantly accumulates and persists for many months following exposure and alters numbers of neurons in the dentate gyrus of the hippocampus and thalamus [5, 6].

In 2008, an ecological birth cohort assessment of Thimerosal exposure in infants and neurodevelopment disorders within the computerized medical records of the Vaccine Safety Datalink (VSD) database was undertaken [7]. A total of 278,624 subjects were examined in birth cohorts from 1990–1996 that had received their first oral polio vaccination by 3 months of age in the VSD. The birth cohort prevalence rate of the medically diagnosed pervasive developmental disorders (PDDs) of autism and autism spectrum disorder (ASD) was calculated. Exposures to organic Hg from all Thimerosal-containing vaccines were calculated by birth cohort for specific exposure windows from birth to 7 months and birth to 13 months of age. Poisson regression analysis was used to model the association between the prevalence of outcomes and organic Hg doses from Thimerosal-containing vaccines. That study found consistent significantly increased rate ratios for diagnosed autism and ASD with increasing organic Hg exposure from Thimerosal-containing vaccines from birth to 7 months and birth to 13 months of age.

Subsequently, in 2013, a case-control assessment of organic Hg from Thimerosal-containing hepatitis B vaccination in infants who were diagnosed autism in the computerized medical records within the VSD database was undertaken [8]. Subjects diagnosed with autism in comparison to controls were significantly more likely to have received increased organic Hg exposure from Thimerosal-containing hepatitis B vaccines within the first month of life, the first 2 months of life, and the first 6 months of life.

Finally, and most recently, a case-control study was undertaken to evaluate the potential dose-response relationship between organic Hg exposure from Thimerosal-containing hepatitis B vaccines administered within the first 6 months of life and the risk of diagnosed PDD [9]. On a per microgram (μg) of organic Hg basis, cases diagnosed with a PDD were significantly more likely than controls to have received increased organic Hg from Thimerosal-containing hepatitis B vaccines administered within the first 6 months of life.

The purpose of the present study was to examine the consistency and extend the previously mentioned studies in the VSD database by undertaking a case-control study to examine organic Hg exposure from the administration of Thimerosal in *Haemophilus influenzae* type b (Hib)-containing vaccines at specific times within the first 15 months of life among subjects diagnosed with a PDD in comparison to controls.

## Methods

The study protocol employed was approved by the US Centers for Disease Control and Prevention (CDC), the Institutional Review Board (IRB) of Kaiser Permanente North-West (KPNW), and the IRB of Kaiser Permanente Northern California (KPNC). The data were analyzed at the secure Research Data Center of the National Center for Health Statistics in Hyattsville, MD. The views expressed in this study do not necessarily reflect those of the CDC or those of Kaiser Permanente.

### Determining the Population at Risk

A cohort of over 1.95 million children enrolled in the VSD project (updated through the end of 2000) from KPNW, Kaiser Permanente Colorado (KPC), and KPNC were examined using SAS® software. The VSD project was created in 1991 by the National Immunization Program (NIP) of the CDC [10–12]. The project links medical event information, specific vaccine history, and selected demographic information from the computerized databases of several Health Maintenance Organizations (HMOs). The cohort examined was comprised of individuals with non-missing date of birth and non-missing gender, who were continuously HMO enrolled from their date of birth.

### Determining Cases

The outcome files (inpatient and outpatient diagnoses) from this population were then reviewed to find the first instance of International Classification of Disease, 9th revision (ICD-9) diagnosed PDD (ICD-9 code: 299.xx). If there were multiple instances of the same diagnosis in a child, only the first instance was counted. All subjects diagnosed with PDD had to be continuously HMO enrolled from birth until their initial PDD diagnosis. In addition, only subjects diagnosed with a PDD following the administration of all the vaccines under study were included in the present analyses as cases. A total of 534 cases diagnosed with PDD (males = 432, females = 102, male/female ratio = 4.2:1) born between 1991 and 2000 were identified. These subjects diagnosed with PDD were evaluated to determine their age of initial diagnosis of PDD (mean ± standard deviation = 4.1 ± 1.56 years old).

In addition, the generally accepted biologically non-plausible linkage between Thimerosal exposure and subsequent diagnosis of febrile seizure was examined as a control outcome. The outcome files (inpatient and outpatient diagnoses) from this population were reviewed to find the first instance of febrile seizures for each subject defined as 780.3 by ICD-9 coding. If there were multiple instances of the same diagnosis in a subject, only the first instance was counted. In addition, only subjects diagnosed with febrile seizures following the administration of all the vaccines under study were included in the present analyses as cases. A total of 5886 cases diagnosed with febrile seizures (males = 3305, females = 2581, male/female ratio = 1.3:1) born between 1991 and 2000 were identified. These subjects diagnosed with febrile seizures were evaluated to determine their age of initial diagnosis of febrile seizures (mean ± standard deviation = 1.54 ± 1.3 years old).

### Determining Controls

In order to identify controls without a PDD diagnosis who would have had only a minimal chance of receiving such a diagnosis, controls had to have been continuously enrolled from birth for at least 7.22 years (mean age of initial diagnosis of PDD +2× standard deviation of mean age of initial diagnosis of PDD). Applying this follow-up criterion yielded a total of 25,632 controls without a PDD diagnosis (males = 13,110, females = 12,522, male/female ratio = 1.05) born between 1991 and 1993.

In order to identify controls without a febrile seizure diagnosis who would have had only a minimal chance of receiving such a diagnosis, controls had to have been continuously enrolled from birth for at least 4.14 years (mean age of initial diagnosis of febrile seizure +2× standard deviation of mean age of initial diagnosis of febrile seizure). Applying this follow-up criterion yielded a total of 85,111 controls without febrile seizure diagnosis (males = 43,615, females = 41,496, male/female ratio = 1.05) born between 1991 and 1996.

### Thimerosal in Hib-Containing Vaccine Exposure

The vaccine file for cases and controls was then reviewed to determine the exact dates of Hib-containing vaccine administration. Overall, among the cases and controls, Hg exposure was assigned as follows: 25-μg organic Hg per dose for whole-cell diphtheria-tetanus-pertussis (DTwP)-Hib vaccine; 25-μg organic Hg per dose for acellular diphtheria-tetanus-pertussis (DTaP)-Hib vaccine; 25-μg organic Hg per dose for Hib vaccine manufactured by Lederle, Praxis Biologics, Wyeth-Ayerst, or Aventis Pasteur, Inc.; 0-μg organic Hg per dose for Hib vaccine manufactured by Merck and Company, Inc. or GlaxoSmithKline; 0-μg organic Hg per dose for Hib-hepatitis B vaccine; and 0-μg organic Hg per dose for those not receiving any type of Hib-containing vaccine. The Thimerosal content for each of the aforementioned vaccines was determined from the official report by the Committee on Infectious Diseases and Committee on Environmental Health of the American Academy of Pediatrics [13].

### Statistical Analyses

The Fisher’s exact test statistic and logistic regression statistic from StatsDirect (Version 2.8.0) were utilized for statistical analyses, and a two-sided *p* value <0.05 was considered statistically significant. In the first case-control experimental group (experiment I), the data was examined to determine the frequency of exposure to 50-μg organic Hg from Thimerosal in Hib-containing vaccines in the first 4 months of life in comparison to the frequency to 25-μg organic Hg from Thimerosal in Hib-containing vaccines in the first 4 months of life among cases diagnosed with PDD and controls using the Fisher’s exact test statistic. In the second case-control experimental group (experiment II), the data was examined to determine the frequency receiving 75-μg organic Hg Thimerosal in Hib-containing vaccines within the first 6 months of life in comparison to the frequency receiving 25-μg organic Hg from Thimerosal in Hib-containing vaccines in the first 6 months of life among cases and controls using the Fisher’s exact test statistic. In the third case-control experimental group (experiment III), the data was examined to determine the frequency of receiving 100-μg organic Hg Thimerosal Hib-containing vaccines within the first 15 months of life in comparison to the frequency of receiving 25-μg organic Hg in the first 15 months of life from Thimerosal Hib-containing vaccines among cases and controls using the Fisher’s exact test statistic. The aforementioned experiments were repeated when separating the data so that male cases diagnosed with PDD were compared to male controls (experiments IV–VI) and female cases diagnosed with PDD were compared to female controls (experiments VII–IX). In addition, the aforementioned experiments were repeated for the control outcome of febrile seizure for the diagnosis among cases in comparison to controls (experiments X–XII) and by comparing cases diagnosed with PDD to controls with a reduced length of follow-up (4.11 years in comparison to 7.22 years) (experiments XIII–XV). Finally, the logistic regression statistics were utilized to determine, among cases diagnosed with PDD in comparison to controls and among cases diagnosed with febrile seizures in comparison to controls, the frequency of receiving increasing doses of organic Hg from Thimerosal in Hib-containing vaccines administered within the first 15 months of life.

## Results

Table 1 displays the relationship between cases diagnosed with PDD and controls being administered increasing doses of organic Hg from Thimerosal in Hib-containing vaccines at several specific points within the first 15 months of life. Overall, it was observed that cases diagnosed with PDD were significantly more likely to receive 75-μg organic Hg exposure from Thimerosal in Hib-containing vaccines compared to 25-μg organic Hg exposure from Thimerosal in Hib-containing vaccines administered within the first 6 months of life (odds ratio = 1.97, *p* < 0.001) and significantly more likely to receive 100-μg organic Hg exposure from Thimerosal in Hib-containing vaccines in compared to 25-μg organic Hg exposure from Thimerosal in Hib-containing vaccines administered within the first 15 months of life (odds ratio = 3.94, *p* < 0.0001) than controls.

**Table 1:** A summary of organic Hg exposure from Thimerosal in Hib-containing vaccines among cases diagnosed with pervasive developmental disorders in comparison to controls within the VSD database

Group examined	Cases with a pervasive developmental disorder diagnosis (%)	Controls without a pervasive developmental disorder diagnosis^1^ (%)	Odds ratio (95 % CI)	*p* value
Experiment I				
50-μg organic Hg within the first 4 months	101 (63.52)	3018 (63.71)		
			1 (0.71–1.4)	>0.99
25-μg organic Hg within the first 4 months	58 (36.48)	1719 (36.29)		
Experiment II				
75-μg organic Hg within the first 6 months	74 (65.49)	2310 (49.02)		
			1.97 (1.31–3)	<0.001
25-μg organic Hg within the first 6 months	39 (34.51)	2402 (50.98)		
Experiment III				
100-μg organic Hg within the first 15 months	82 (75.93)	1863 (44.46)		
			3.94 (2.49–6.41)	<0.0001
25-μg organic Hg within the first 15 months	26 (24.07)	2327 (55.54)		

^1^Controls were enrolled from birth until at least 7.22 years old without a pervasive developmental disorder diagnosis

Tables 2 and 3, respectively, display the relationship between male cases diagnosed with PDD and male controls and female cases diagnosed with PDD and female controls being administered increasing doses of organic Hg exposure from Thimerosal in Hib-containing vaccines at several specific points within the first 15 months of life. Male cases diagnosed with PDD were significantly more likely to receive 75-μg organic Hg exposure from Thimerosal in Hib-containing vaccines in comparison to 25-μg organic Hg exposure from Thimerosal in Hib-containing vaccines administered within the first 6 months of life (odds ratio = 2.29, *p* < 0.0005) and significantly more likely to receive 100-μg organic Hg exposure from Thimerosal in Hib-containing vaccines compared to 25-μg organic Hg exposure from Thimerosal in Hib-containing vaccines administered within the first 15 months of life (odds ratio = 3.98, *p* < 0.0001) than male controls. By contrast, female cases diagnosed with PDD were only significantly more likely to receive 100-μg organic Hg exposure from Thimerosal in Hib-containing vaccines in comparison to 25-μg organic Hg exposure from Thimerosal in Hib-containing vaccines administered within the first 15 months of life (odds ratio = 3.54, *p* < 0.005).

**Table 2:** A summary of organic Hg exposure from Thimerosal in Hib-containing vaccines among male cases diagnosed with pervasive developmental disorders in comparison to male controls within the VSD database

Group examined	Male cases with a pervasive developmental disorder diagnosis (%)	Male controls without a pervasive developmental disorder diagnosis^1^ (%)	Odds ratio (95 % CI)	*p* value
Experiment IV				
50-μg organic Hg within the first 4 months	82 (63.08)	1584 (63.9)		
			0.96 (0.66–1.42)	>0.80
25-μg organic Hg within the first 4 months	48 (36.92)	895 (36.10)		
Experiment V				
75-μg organic Hg within the first 6 months	60 (68.18)	1175 (48.33)		
			2.29 (1.43–3.75)	<0.0005
25-μg organic Hg within the first 6 months	28 (31.82)	1256 (51.67)		
Experiment VI				
100-μg organic Hg within the first 15 months	63 (76.83)	981 (45.44)		
			3.98 (2.33–7.09)	<0.0001
25-μg organic Hg within the first 15 months	19 (23.17)	1178 (54.56)		

^1^Controls were enrolled from birth until at least 7.22 years old without a pervasive developmental disorder diagnosis

**Table 3:** A summary of organic mercury exposure from Thimerosal in Hib-containing vaccines among female cases diagnosed with pervasive developmental disorders in comparison to female controls within the VSD database

Group examined	Female cases with a pervasive developmental disorder diagnosis (%)	Female controls without a pervasive developmental disorder diagnosis^1^ (%)	Odds ratio (95 % CI)	*p* value
Experiment VII				
50-μg organic Hg within the first 4 months	19 (65.52)	1434 (63.51)		
			1.09 (0.48–2.64)	>0.99
25-μg organic Hg within the first 4 months	10 (34.48)	824 (36.49)		
Experiment VIII				
75-μg organic Hg within the first 6 months	14 (56)	1135 (49.76)		
			1.29 (0.54–3.14)	>0.50
25-μg organic Hg within the first 6 months	11 (44)	1146 (50.24)		
Experiment IX				
100-μg organic Hg within the first 15 months	19 (73.08)	882 (43.43)		
			3.54 (1.41–9.99)	<0.005
25-μg organic Hg within the first 15 months	7 (26.92)	1149 (56.57)		

^1^Controls were enrolled from birth until at least 7.22 years old without a pervasive developmental disorder diagnosis

Table 4 evaluates the relationship between cases diagnosed with the control outcome of febrile seizure and controls being administered increasing doses of organic Hg from Thimerosal in Hib-containing vaccines at several specific points within the first 15 months of life. Among cases diagnosed with febrile seizure in comparison to controls, there was no increased frequency of exposure to increasing dosing of organic Hg from Thimerosal in Hib-containing vaccines administered at any of the specific periods examined within the first 15 months of life.

**Table 4:** A summary of organic Hg exposure from Thimerosal in Hib-containing vaccines among cases diagnosed with a febrile seizure in comparison to controls within the VSD database

Group examined	Cases with a febrile seizure diagnosis (%)	Controls without n febrile seizure diagnosis^1^ (%)	Odds ratio (95 % CI)	*p* value
Experiment X				
50-μg organic Hg within the first 4 months	1215 (62.12)	17,176 (63.54)		
			0.94 (0.86–1.03)	>0.20
25-μg organic Hg within the first 4 months	741 (37.88)	9857 (36.46)		
Experiment XI				
75-μg organic Hg within the first 6 months	936 (62.61)	13,412 (70.5)		
			0.70 (0.63–0.78)	<0.0001
25-μg organic Hg within the first 6 months	559 (37.39)	5613 (29.50)		
Experiment XII				
100-μg organic Hg within the first 15 months	1224 (78.82)	14,402 (78.47)		
			1.02 (0.9–1.16)	>0.75
25-μg organic Hg within the first 15 months	329 (21.18)	3952 (21.53)		

^1^Controls were enrolled from birth until at least 4.14 years old without a febrile seizure diagnosis

Table 5 reveals the potential dose-dependent relationship between cases diagnosed with PDD in comparison to controls and cases diagnosed with febrile seizure in comparison to controls for increasing organic Hg doses from Thimerosal in Hib-containing vaccines administered within the first 15 months of life. On a per microgram of organic Hg basis, cases diagnosed with a PDD in comparison to controls were at significantly greater odds (odds ratio = 1.0197, *p* < 0.0001) of receiving increasing organic Hg exposure from Thimerosal in Hib-containing vaccines. As a consequence for the maximum dose of an additional 75-μg organic Hg exposure from Thimerosal in Hib-containing vaccines administered within the first 15 months of life, cases diagnosed with PDD in comparison to controls were 2.478-fold significantly more likely to receive 100-μg organic Hg from Thimerosal in Hib-containing vaccines in comparison to 25-μg organic Hg exposure from Thimerosal in Hib-containing vaccines administered within the first 15 months of life. By contrast, on a per microgram of organic Hg basis, cases diagnosed with febrile seizure in comparison to controls were no more likely (odds ratio = 0.999, *p* > 0.20) of receiving increasing organic Hg exposure from Thimerosal in Hib-containing vaccines.

**Table 5:** A summary of the potential dose-dependent relationship between exposure to organic Hg from Thimerosal in Hib-containing vaccines administered within the first 15 months of life among cases in comparison to controls

Group examined (ICD-9 Code)	25-μg organic Hg (%) [Reference dose]	50-μg organic Hg (%)	75-μg organic Hg (%)	100-μg organic Hg (%)	Odds ratio per μg organic Hg (95 % CI) [*p* value]
Pervasive developmental disorder cases (299.xx)	26 (6.48)	28 (6.98)	265 (66.09)	82 (20.45)	
					1.0197 (1.0143–1.0251) [<0.0001]
Controls	2327 (15.87)	1960 (13.36)	8517 (58.07)	1863 (12.70)	
Febrile Seizure Cases (315.xx)	329 (6.53)	463 (9.19)	3022 (59.98)	1224 (24.3)	
					0.999 (0.977–1.001) [>0.20]
Controls	3952 (6.04)	4297 (6.57)	42,746 (65.36)	14,402 (22.02)	

Table 6 summarizes the discrete point odds ratio estimates and logistic regression odds ratio estimates for an additional 25-, 50-, or 75-μg organic Hg from Thimerosal in Hib-containing vaccines administered within the first 15 months of life among cases diagnosed with a PDD in comparison to controls. The discrete point odds ratio estimates and logistic regression odds ratio estimates were of similar order-of-magnitude for increasing exposure to organic Hg for cases diagnosed with a PDD in comparison to controls.

**Table 6:** A summary of exposure to organic Hg from Thimerosal in Hib-containing vaccines administered within the first 15 months of life for the cases diagnosed with PDD and controls by both the discrete point odds ratio estimates and the logistic regression odds ratio estimates^1^

		Organic Hg exposure level above the reference dose^1^		
Group examined (ICD-9 Code)	Statistical approach used	25-μg organic Hg (95 % CI)	50-μg organic Hg (95 % CI)	75-μg organic Hg (95 % CI)
	Discrete point odds ratio estimates	1.28 (0.72–2.3)	2.8 (1.9–4.4)	3.9 (2.5–6.4)
Pervasive developmental disorder cases (299.xx)				
	Logistic regression odds ratio estimates	1.493 (1.358–1.628)	1.985 (1.715–2.255)	2.478 (2.073–2.883)

^1^The exposure “reference dose” is 25 μg of organic Hg from Thimerosal in Hib-containing vaccines

## Discussion

As previously published by investigators from the CDC and the US Food and Drug Administration (FDA), products, such as vaccines, which are intended for healthy people, must be held to a high standard of safety assurance [14]. However, these previous investigators described that the study of vaccine risks is more complex than for therapeutic products because the exposure is nearly universal for many vaccines, ensuring the chance occurrence of many subsequent temporally associated adverse outcomes after the administration of vaccines. As a result, these investigators described using the VSD, a consortium of HMOs, to more rigorously evaluate post-vaccination associated risks (hypothesis testing).

The present study revealed cases diagnosed with PDD were overall, among both male and female cases analyzed separately, and on a dose-dependent basis, significantly more likely than controls to receive increased organic Hg exposure from Thimerosal in Hib-containing vaccines administered at specific intervals within the first 15 months of life. The results of the present study are consistent with and extend the previous ecological birth cohort study [7] and case-control studies [8, 9] of the potential relationship between organic Hg exposure from Thimerosal-containing childhood vaccines and PDD in the VSD database. The size and magnitude of the observed relationship on a per microgram organic Hg basis are similar to the previously discussed VSD studies and suggest that the phenomena observed in the VSD database is more than mere chance. In addition, the significant association observed between organic Hg exposure and subsequently diagnosed PDD is consistent with several other studies examining other databases such as the National Health Interview Survey (NHIS) [15] and the Vaccine Adverse Event Reporting System (VAERS) [8, 16].

The results of the present study stand in contrast to six previous epidemiological studies that failed to find a significant relationship between Hg exposure from Thimerosal-containing childhood vaccines and diagnosed PDD [17–22]. As recently reviewed, the aforementioned studies were found to have significant methodological concerns, and, as a consequence, their potential ability to contribute to understanding the potential relationship between organic Hg exposure from Thimerosal-containing childhood vaccines and diagnosed PDD was found to be not interpretable [23].

In further support of the biological plausibility of the phenomena observed in the present study, investigators have examined a number of different lines of scientific inquiry. For example, investigators recently published a critical review on the mechanisms of how limited thiol availability, abnormal sulfation chemistry, and decreased glutathione reserve capacity in children diagnosed with PDD could make them more susceptible to the toxic effects of Thimerosal routinely administered as part of childhood immunization schedules [24]. Another study demonstrated that human neuronal systems exposed to low concentrations of Thimerosal were able to induce cellular damage similar to that observed in the neuropathological studies of subjects diagnosed with a PDD [25]. Finally, several recent studies of Thimerosal exposure in animal model systems resulted in neuropathological and behavioral symptoms similar to those associated with PDD [26–30].

### Strengths/Limitations

In considering the strength of the results observed, the VSD observations made were based upon retrospective assessment of prospectively collected medical records of patients enrolled in various HMOs. As such, the VSD data examined were collected independently of the study design and were, in fact, collected as part of the routine healthcare individuals received as part of the participation with their respective HMOs. Thus, at the times of the collection of these records, the healthcare providers probably were not thinking about the potential association between vaccine exposures and potential subsequent adverse health outcomes.

The study design used to evaluate the potential relationship between exposure and outcome was another significant strength of the present study. Cases examined in the present study had to be enrolled from birth and were required to be continuously enrolled until a medical diagnosis of PDD was made. Moreover, controls had to be enrolled from birth for a sufficient time period to ensure that there was a very small chance that, during additional follow-up, any of the controls would be medically diagnosed with a PDD. As a result, factors associated with enrollment (i.e., adjustment for potential independent variables between cases and controls were not necessary because enrollment was from birth) or healthcare-seeking behavior (i.e., adjustment for potential access/availability of healthcare was continuous among cases and controls) were minimized. In addition, cases diagnosed with a PDD were specifically evaluated to ensure that only those cases diagnosed with an PDD following vaccine administration were considered in the present analyses.

An additional strength of the present study is that it was decided, a priori, to ensure adequate amounts of data for our analyses and that the included controls would be HMO enrolled continuously from birth until they were at least the mean age of initial diagnosis of PDD plus twice the standard deviation of the mean age of initial diagnosis of PDD. For cases diagnosed with a PDD, it was possible to mathematically evaluate the mean and standard deviation of age for initial PDD diagnoses within the VSD. From this information, it was possible to estimate how many additional potential diagnoses of PDD were missed. It was decided, a priori, to ensure adequate amounts of data for our analyses and that controls had to be continuously enrolled in the VSD from birth until they were at least 7.22 years old (mean age of initial diagnosis of PDD +2× standard deviation of the mean age of initial diagnosis of PDD). Based on the data for age of initial diagnosis for PDD, this was a sufficient period to ensure that, with further follow-up, those controls without PDD would probably not receive a PDD diagnoses in the VSD (mathematically there is a <2.5 % chance of these individuals being diagnosed with a PDD with additional follow-up time beyond 7.22 years). As shown in Table 7, when the length of follow-up of controls examined was reduced to only the mean age of initial diagnosis of PDD (i.e., cases/controls were followed for same length of time and mathematically there is an about 50 % error), the overall adverse effects observed on a per microgram basis from increasing exposure to organic Hg exposure from Thimerosal in Hib-containing vaccines completely disappear. Clearly, these analyses establish the importance of following controls in any study of PDDs for a sufficient period to ensure that the risk of a control being subsequently diagnosed with a “case” condition is as small as possible, subject to the limitations of the records that are accessible for study.

**Table 7:** A summary of organic Hg exposure from Thimerosal in Hib-containing vaccines among cases diagnosed with a PDD in comparison to controls with a reduced length of follow-up^1^

Group examined	Cases with a pervasive developmental disorder diagnosis (%)	Controls without a pervasive developmental disorder diagnosis (%)	Odds ratio (95 % CI)	*p* value
Experiment XIII				
50 μg organic Hg within the first 4 months	101 (63.52)	17,938 (63.60)		
			1 (0.72–1.38)	>0.95
25-μg organic Hg within the first 4 months	58 (36.48)	10,268 (36.40)		
Experiment XIV				
75-μg organic Hg within the first 6 months	74 (65.49)	14,027 (70.41)		
			0.8 (0.54–1.18)	>0.20
25-μg organic Hg within the first 6 months	39 (34.51)	5896 (29.59)		
Experiment XV				
100-μg organic Hg within the first 15 months	82 (75.93)	15,020 (78.41)		
			0.87 (0.56–1.35)	>0.50
25-μg organic Hg within the first 15 months	26 (24.07)	4135 (21.59)		

^1^Controls were enrolled from birth until at least 4.11 years old without a pervasive developmental disorder diagnosis

A further strength of the present study was the specific methods employed to evaluate differences in cumulative doses of organic Hg received at specific intervals during the infant period were not the result of small group of children receiving anomalous exposure to vaccines. Instead, the subjects examined were exposed to different levels of organic Hg based upon differences in the Thimerosal content of licensed Hib vaccines [13] and the recommendations on the ages for the administration of Hib vaccines to children made by the 1991 Advisory Committee on Immunization Practices recommendations [31].

However, the results of the present study may have a number of potential limitations. For example, the results observed may have occurred from unknown biases or cofounders present in the datasets examined. This seems unlikely because the control outcome of febrile seizure (i.e., an outcome that is not biologically plausibly linked to postnatal organic Hg exposure from Thimerosal-containing vaccines) was examined, using the same methodology employed for PDD. As shown in Tables 4 and 5, no similar patterns of significant associations were observed as those that were found for organic Hg exposure from Thimerosal in Hib-containing vaccines and the subsequent risk of PDD diagnosis.

Another potential limitation of the present study is that the results observed may be the result of statistical chance. However, such a possibility would be unlikely given the limited number of statistical tests performed, the highly significant results observed (most *p* values observed were <0.01), and the consistency in the direction and magnitude of the results observed. In addition, it was observed that there were similar order-of-magnitude odds ratios for increasing organic Hg exposure among cases diagnosed with a PDD in comparison to controls for discrete point estimates and logistic regression estimates.

Still, other potential limitations of the present study include the possibilities that some of the individuals in VSD examined may have had more subtle neurological dysfunction, which was not brought to the attention of their healthcare providers; healthcare providers may have misdiagnosed some individuals; or some vaccine exposures may not have been appropriately classified. In addition, it is possible that some of the organic Hg exposures from the Hib-containing vaccines were different than the estimated amount. For example, ActHIB vaccine manufactured by Aventis Pasteur was always Thimerosal free, but it was usually reconstituted with Thimerosal-containing Tripedia, but it is possible that some doses were reconstituted with just plain saline. Similarly, some Hib vaccines by Merck and Company, Inc. may have had 12.5-μg organic Hg per dose, and not the assigned 0-μg organic Hg per dose. In addition, it is possible that some very small number of doses of HibTITTER vaccine manufactured by Lederle may have come in single-dose vials with 0-μg organic Hg per dose, and not the assigned 25-μg organic Hg per dose. These limitations, while possibly present in the data examined in the current study, should not have significantly impacted the results observed because it is unclear how differential application would have occurred to the study cohorts examined based upon the Thimerosal doses that the individuals received. Moreover, misclassification occurring in the data examined would tend to bias any results observed toward the null hypothesis, since such effects would result in individuals being placed in the wrong exposure and/or outcome categories examined, and result in decreased statistical power to determine true potential exposure-outcome relationships.

In addition, another potential limitation of the present study is that exposures to other sources of Hg were not evaluated. At this time, data analysis restrictions imposed by the CDC prevent us from studying more than one type of vaccine subjects received, despite the fact that it is very likely that the subjects examined incurred other organic Hg exposures from other Thimerosal-containing childhood vaccines. It is also likely that subjects examined received Hg exposures from dental amalgams, fish, or other environmental sources. While all these other sources of Hg may play a significant involvement in the pathogenesis PDD, these unaccounted for Hg exposures would actually tend to bias the results observed toward the null hypothesis because they potentially would confound the specific exposure classifications of Hg examined. For example, individuals classified as having lower organic Hg exposure from Thimerosal-containing vaccines may have actually received high doses of Hg from other sources, and individuals having higher organic Hg exposure from Thimerosal-containing vaccines may have actually received low doses of Hg from other sources, with the net result tending to minimize the magnitude of the associations observed.

It is also possible that the findings reported may be the result of other components of the vaccines studied, which in isolation or synergistically interacted with the organic Hg exposures examined in the present study. These possibilities seem remote, since multiple different brands of Hib-containing vaccines were examined (with many different amounts/types of adjuvants and trace constituents), but a significant overall and dose-dependent relationship was observed among cases diagnosed with a PDD in comparison to controls for increasing organic Hg exposure. Moreover, any effects of other components of vaccines working in isolation or synergistically would tend to bias the results observed toward the null hypothesis because they were not considered in the statistical analyses undertaken.

Additionally, a potential limitation of the present study is there may be differences in the general health status among the subjects examined independent of exposure to organic Hg from Hib-containing vaccine administration. As described previously by investigators from the CDC [32], confounding of this sort is a general problem for studies of adverse reactions to prophylactic interventions, as they may be withheld from some individuals precisely because they are already at high risk of the adverse event, and, as a consequence, studies that fail to adequately control for such confounding factors are likely to underestimate the risk of adverse events attributable to vaccination. In order to help minimize that potential effect in our analyses, we utilized a reference group that received 25 μg organic Hg exposure from Hib-containing vaccines in all of our statistical analyses. Despite our attempt to minimize these phenomena in our analyses, we did observe that there was an apparent protective association for increased organic Hg exposure from Thimerosal in Hib-containing vaccines for our control outcome of febrile seizure compared to controls. As a consequence, there was a “healthy vaccine” effect in our data. Since this phenomenon is apparent in the data examined, the significantly increased positive dose-dependent associations observed for the PDD probably underestimates the true extent of the relationship between organic Hg exposure from Thimerosal in Hib-containing vaccines and the subsequent PDD outcome studied.

Finally, the current study suffers from the potential limitation that analyses were not conducted to further explore the precise timing and cumulative doses of organic Hg from all Thimerosal-containing childhood vaccines associated with maximum adverse consequences. In future studies, it would be worthwhile to further explore these precise-timing and cumulative-doses phenomena. In addition, it would be valuable to evaluate other neurodevelopmental outcomes, as well as other covariates such as gender, race, birth weight, etc., that may further affect the magnitude of the adverse effects found.

## Conclusion

The present case-control study revealed consistent and significantly increased associations between exposure to organic Hg from Thimerosal in Hib-containing vaccines administered at specific times within the first 15 months of life among subjects (both male and female) subsequently diagnosed with a PDD in comparison to controls. The results also revealed that, on a dose-dependent basis, subjects diagnosed with a PDD were significantly more likely than controls to have received increased organic Hg exposure from Thimerosal in Hib-containing vaccines. The present study is both consistent with and extends previous epidemiological studies in the VSD database and provides compelling new epidemiological evidence supporting a significant relationship between increasing organic Hg exposure from Thimerosal-containing childhood vaccines and the subsequent risk of a PDD.

As previously described, routine childhood vaccination may be an important public health tool to reduce the morbidity and mortality associated with infectious diseases [33]. However, it is also a public health imperative to end the unnecessary addition of organic Hg to vaccines in the form of Thimerosal used as a preservative or otherwise, based on data showing an association between its administration and the increasing risk for the adverse outcomes studied.
